# Gender Differences in Compulsive Buying Disorder: Assessment of Demographic and Psychiatric Co-Morbidities

**DOI:** 10.1371/journal.pone.0167365

**Published:** 2016-12-01

**Authors:** Cristiana Nicoli de Mattos, Hyoun S. Kim, Marinalva G. Requião, Renata F. Marasaldi, Tatiana Z. Filomensky, David C. Hodgins, Hermano Tavares

**Affiliations:** 1 Impulse Control Disorders Outpatient Unit, Institute of Psychiatry, University of Sao Paulo, São Paulo, Brazil; 2 Addictive Behaviours Laboratory, University of Calgary, Calgary, Canada; Ariel University, ISRAEL

## Abstract

Compulsive buying is a common disorder found worldwide. Although recent research has shed light into the prevalence, etiology and clinical correlates of compulsive buying disorder, less is known about gender differences. To address this empirical gap, we assessed potential gender differences in demographic and psychiatric co-morbidities in a sample of 171 compulsive buyers (20 men and 151 women) voluntarily seeking treatment in São Paulo, Brazil. A structured clinical interview confirmed the diagnosis of compulsive buying. Of the 171 participants, 95.9% (*n* = 164) met criteria for at least one co-morbid psychiatric disorder. The results found that male and female compulsive buyers did not differ in problem severity as assessed by the Compulsive Buying Scale. However, several significant demographic and psychiatric differences were found in a multivariate binary logistic regression. Specifically, male compulsive buyers were more likely to report being non-heterosexual, and reported fewer years of formal education. In regards to psychiatric co-morbidities, male compulsive buyers were more likely to be diagnosed with sexual addiction, and intermittent explosive disorder. Conversely, men had lower scores on the shopping subscale of the Shorter PROMIS Questionnaire. The results suggest that male compulsive buyers are more likely to present with co-morbid psychiatric disorders. Treatment planning for compulsive buying disorder would do well to take gender into account to address for potential psychiatric co-morbidities.

## Introduction

Compulsive buying disorder (CBD) is characterized by excessive shopping behavior that results in distress and causes marked interpersonal and financial difficulties [1]. CBD is a common disorder worldwide, with estimates ranging from 6–7% [2]. While CBD was first described by Kraepelin [3] and Bleuler [4] at the beginning of 20th century, it is only recently that empirical investigations have begun delineating the characteristics, prevalence and clinical correlates associated with compulsive buying. Despite the growing empirical literature on CBD, little is known about its potential gender differences. This is a gap in the literature as gender differences between male and female compulsive buyers may have important clinical and treatment implications.

To date, only a handful of empirical studies have examined gender differences in CBD—with mixed results regarding the rates of compulsive buying in men and women. For example, in a German sample of community participants [5], rates of compulsive buying were found to occur equally between men and women. Conversely, however, higher rates of compulsive buying have been observed in community samples from United Kingdom [6] and Spain [7]. Furthermore, 80% to 94% of compulsive buyers who seek treatment are women [8,9]. That said, it has been argued that the gender differences in treatment seeking may not reflect true prevalence differences but it is merely an artifact of women being more likely to recognize having a problem with their shopping behavior, whereas men tend to see their compulsive buying as "collecting"[10].

Gender differences, however, have been observed in regards to the objects purchased. Women tend to purchase clothes, purses, shoes, perfumes, make up and jewelry; while men prefer electronics, suits, clocks and cars [8,11,12,13]. The aim of the present research was to add to the existing literature by examining the potential gender differences in compulsive buying severity, demographics and psychiatric co-morbidities in a treatment seeking sample of compulsive buyers. Due to the paucity of empirical research in this domain, we were hesitant to make any strong a priori hypotheses. As such, the results of the present research should be viewed as preliminary evidence of the potential gender differences in CBD.

## Methods

### Participants and Procedure

The participants were 171 compulsive buyers voluntarily seeking treatment at specialized Health Service in São Paulo, Brazil from 2015–2016. Our center is the largest (if not the only) specialized treatment center for Impulse Control Disorders in São Paulo, and thus is well-known. Individuals wishing to seek treatment self-refer by calling the treatment center. As such, all participants first sought treatment. During this call, individuals are screened using the Portuguese adapted version of the Compulsive Buying Scale [14]. Patients whose scores indicate a possible presence of CBD are then provided an appointment time with a registered psychiatrist specializing in impulse control disorders to confirm a diagnosis of CBD through a Impulse Control Disorder structured clinical interview modeled after the Structured Clinical Interview for DSM (ICD-SCID). As per the protocol at the treatment center, a diagnosis of CBD is then confirmed by a registered psychologist, who also collects additional measures on compulsive buying behaviors. Eligible patients are provided with information about research participation in a variety of projects, and a written informed consent is obtained from all patients who indicate their willingness to participate. Patients are clearly informed that treatment is not contingent on research participation. Treatment, provided on an outpatient basis, consists of 24 sessions (4 sessions of Motivational Interviewing and 20 sessions of group Cognitive Behavioral Therapy).

### Ethics Statement

The sample for this study was drawn from two research projects: a clinical trial approved by the Brazilian national research ethics committee (CEP/CONEP) (#32270214.9.0000.0065), and registered at ClinicalTrials.gov (#NCT02138058) and a study examining a dimensional model of impulsivity, which obtained ethics approval at the senior author’s home institution (CEP/CONEP) (#12820813.5.0000.0068). The clinical investigation was conducted according to the principles expressed in the Declaration of Helsinki. No specific ethical approval was obtained for the present manuscript, as the results presented herein were a secondary analysis of the aforementioned studies.

The data underlying this present research will be made available upon request. To request data, please email Faculty of Medicine at University of Sao Paulo’s Ethics Board (email: cappesq@hcnet.usp.br). We have decided to not upload the data onto a repository, as this was not communicated to the participants prior to data collection.

### Measures

#### Demographics

A standard demographic questionnaire used at the specialized health service collected standard demographic information. The questionnaire included items such as, gender, age, ethnicity, sexual orientation, education, employment status, income and religion.

#### Compulsive Buying Severity

Compulsive buying severity was assessed using the Portuguese adapted version of the Compulsive Buying Scale (CBS) [14]. The original CBS was developed in 1992 by Faber and O’Guinn [1] and is one of the most widely used measure of compulsive buying severity. The scale contains 7 items, anchored from 1 to 5 and assesses problems associated with compulsive buying, including: loss of control with finances, symptoms of withdrawal when not shopping and shame about one’s shopping. The authors created a scoring system using a regression equation with item weightings. It was determined that a cut off score of -1.34 was sensitive in identifying CBD, with lower scores indicating greater compulsive buying severity.

The CBS was adapted into Portuguese through several iterations, including back translations by professional translators and a psychologist [14]. Thereafter, participants from the general population were recruited for language adjustments. The Portuguese version of the CBS showed adequate psychometric properties when tested with non-clinical and clinical populations.

#### Mental Health

Co-morbid mental health was assessed using the validated Portuguese version of the Mini International Neuropsychiatric Interview (M.I.N.I) [15]. The M.I.N.I is a brief structured interview that assesses mood and anxiety disorders, obsessive compulsive disorder, post-traumatic stress disorder, psychosis and substance use disorders. The M.I.N.I has been shown to demonstrate strong psychometric properties when compared to other structured clinical interviews, such as the SCID [16]. The ICD-SCID, a semi-structured interview modeled after the SCID assessed for impulse control disorders (kleptomania, explosive intermittent disorder, excoriation disorder and trichotillomania) and behavioral addictions (internet addiction and sexual addiction).

#### Addictive Behaviors

A Portuguese version of the Shorter PROMIS Questions (SPQ) [17] was used to assess 16 domains of addictive behaviors: alcohol, caffeine, compulsive helping (dominant), compulsive helping (submissive), drugs, exercise, food binging, food starving, gambling, tobacco, prescribed drugs, relationship (dominant), relationship (submissive), sex, shopping and work. Each domain consisted of 10 items anchored from 0 (*not like me*) to 5 (*like me*). Each subscale is summed on a continuous score with a minimum score of 0 to maximum score of 50, with higher scores indicating greater problem severity with the addictive behavior. The items assess behaviors (e.g., *“I have often bought so many goods [groceries*, *sweets*, *household goods*, *books*, *etc*.*] that it would take a month to get through them”*) and attitudes (e.g., *“I have prided myself on the speed with which I can get to have sex with someone and I have found that sex with a complete stranger is stimulating”*) related to the addictive behaviors. For a full list of the items per each addictive behavior, see Christo et al [17].

The SPQ was developed due to empirical evidence and clinical observations of the high co-morbidity between addictive disorders, including behavioral addictions (e.g., gambling) [18]. Indeed, according to the syndrome model of addiction, all addictive disorders share an underlying cause, with the different expressions of an addictive disorder manifesting based on the individuals environment [19]. Given that addictive disorders share an underlying cause, they are likely to be co-morbid. Thus, it is beneficial to measure an array of addictive behaviors in individuals. The SPQ was developed with this goal in mind [17]. The validation of the SPQ was done with clinical samples, and using a cutoff of 90^th^ percentile demonstrates excellent sensitivity [17].

## Data Analysis

A multi-step analytic approach was utilized. First, frequencies, means and standard deviations provided descriptive characteristics of the sample. To assess for gender differences, univariate analyses were conducted. Chi-square tests were used for categorical variables and t-tests were conducted on continuous variables. Fisher’s Exact tests was used for categorical variables when expected cell counts were less than five. Mann-Whitney U non-parametric test was used when the assumption of normality was violated for continuous measures. The final step assessed the independent contribution of variables found to be significant at the bivariate level. A multivariate backwards-binary logistic regression was conducted with gender (0 = female, 1 = male) as the dependent variable and variables that were associated with *p* < .10 in the univariate analysis entered as the predictor variables [20].

## Results

### Preliminary Analysis

#### Sample Characteristics

The sample consisted of 20 males (11.7%), and 151 females (88.3%) with a mean age of 38.71 years (*SD* = 10.6) (Table 1). The sample consisted primary of Caucasians (74.1%), with marital status being roughly equal: single (48.1%), married (42.6%). The sample was fairly well educated with an average of 15.05 years of schooling (*SD* = 3.1), corresponding to university education. 72.5% reported being employed and earning an average of $2510USD per month. In terms of religion, 29.8% were Catholic, 27.3% reported spirituality as their religion, 26.7% reporting other and 16.1% were Protestant.

**Table 1:** Demographics and shopping behavior characteristics between male and female compulsive buyers.

	Males (*n* = 20)			Females (*n* = 151)			Test	P
Characteristics	N	%	M(SD)	N	%	M(SD)		
**Age**			41.2 (12.5)			38.38 (10.3)	U = 1.28^a^	0.277
**Ethnic group (color)**								0.775^c^
Whites	13	72.2		107	74.3			
Blacks	1	5.6		13	9.0			
Mixed-Race	4	22.2		21	14.6			
Others	0	0		3	2.1			
**Marital Status**								1.000^c^
Married	8	44.4		61	42.4			
Single	9	50		69	47.9			
Other	1	5.6		14	9.7			
**Sexual Orientation**								<0.001^c^*
Heterossexual	12	66.7		139	96.5			
Homo or bisexual	6	33.7		5	3.5			
**Years of Formal Education**	13.94	2.9		15.18	3.1		U = 0.96^a^	0.068
**Employment Status**								0.286^c^
Employed	12	60		112	74.2			
Unemployed	8	40		39	25.8			
**Income ($USD)**			2168.2 (1189.29)			2553.58 (2357.81)	U = 1.26^a^	0.852
**Religion**								0.578^c^
Catholic	5	27.8		43	30.1			
Protestant	5	27.8		21	14.7			
Spiritism	4	22.2		40	28.0			
Other	4	22.2		39	27.3			
**Compulsive Buying Scale**			-3.61(2.46)			-4.66 (1.60)	U = 964.5^a^	0.114
**Age of onset of compulsive buying**			21.18(8.7)			21.93 (8.6)	U = 1078^a^	0.684
**Where they usually buy**								
Stores/shopping mall	20	100		151	99.3		*χ*^2^ = 0.13^b^	1.000
Catalogue	3	16.7		54	39.4		*χ*^2^ = 3.54^b^	0.071
Internet	11	57.9		106	72.6		*χ*^2^ = 1.76^b^	0.184
Street Vendors	10	55.6		84	58.3		*χ*^2^ = 0.05^b^	0.822
TV or other	2	10		11	7.3		*χ*^2^ = 0.19^b^	0.652

Note

^a^Mann-Whitney U

^b^Chi-Square

^c^Fishers Exact test was used as expected cell counts were less than 5

*p < .001.

In regards to compulsive buying characteristics (Table 1), the mean age of onset was 21.85 years (*SD* = 8.62). 170 (99.4%) reported purchasing items in shopping malls, followed by the internet (*n* = 117; 70.9%), street vendors (*n* = 95; 58%), catalogues (*n* = 57; 36.8%) and TV/other (*n* = 13; 7.6%). Men and women did not differ in scores on the Compulsive Buying Scale, *p* = .11.

### Univariate Analyses

#### Demographics

When comparing male and female compulsive buyers on demographic characteristics (Table 1), the results found that male compulsive buyers (33.7%) were significantly more likely to report their sexual orientation as non-heterosexual than female compulsive buyers (3.5%), *p* < .001. Men and women did not differ significantly in age, ethnicity, marital status, years of education, employment, income, and religion, *ps >* .06.

#### Psychiatric Co-morbidities

As shown in Table 2, co-morbidity with other psychiatric disorders was high, with 164 participants (95.9%) meeting criteria for at least one additional psychiatric diagnosis. Anxiety and mood disorders were the most frequent psychiatric co-morbidity, with 95 (*n* = 55.6%) meeting criteria for a generalized anxiety disorder, followed by major depressive episode (*n* = 91; 53.2%). Two gender differences were uncovered. Male compulsive buyers were significantly more likely to present with a co-morbid sexual addiction (40% vs. 8.2%), *p* < .001 and intermittent explosive disorder (20% vs. 5.3%), *p* = .037. No significant differences emerged between the genders in regards to other diagnoses, *ps* > .11.

**Table 2:** Psychiatric co-morbdity and gender diferences in compulsive buyers.

	Males (*n =* 20)		Females (*n* = 151)		χ^2^	P
Psychiatric Comorbity	*n*	%	*n*	%		
Major Depressive Episode	11	55	80	53	0.029	0.865
Dysthmia	2	10	13	8.6		0.689^d^
Suicidal Risk	9	45	59	39.8	0.259	0.611
a. mild	5	25	45	29.8	0.197	0.657
b.moderate	1	5	3	2		0.395^d^
c.severe	3	15	15	9.9		0.447^d^
Panic Disorder	3	15	40	30.5		0.742^d^
Agoraphobia	2	10	41	27.2	2.760	0.097
Social Phobia	4	20	36	23.8		1.000^d^
PTSD^a^	0	0	9	6		0.601^d^
GAD^b^	11	55	84	55.6	0.003	0.958
OCD^c^	5	25	22	14.6		0.323^d^
Internet Addiction	6	30	28	18.5		0.239^d^
Sexual Addiction	8	40	6	4		<0.001**
Bulimia Nervosa	1	5	13	8.6		1.000^d^
Binge Eating Disorder	4	20	20	13.2		0.490^d^
Kleptomania	0	0	4	2.6		1.000^d^
Intermittment Explosive Disorder	4	20	8	5.3		0.037^d^*
Excoriation Disorder	3	15	24	15.9		1.000^d^
Trichotillomania	1	5	6	4		0.588^d^

^a^ *PTSD—Post Traumatic Stress Disorder*

^b^ GAD—Generalzed Anxiety Disorder

^c^ OCD—Obsessive Compulsive Disorder

^d^Fishers Exact test was used as expected cell counts were less than 5

*p < .05

**p < .001

#### Addictive Behaviors

Compared to female compulsive buyers, male compulsive buyers were more likely to report problems with alcohol use, gambling, sex, and work. Male compulsive buyers also reported higher levels in submissive/dominant helping and higher levels of relationship submissiveness compared to female compulsive buyers. Lastly, the compulsive shopping subscale had the highest mean on the SPQ for both men and women (Table 3).

**Table 3:** Differences between men and women in addictive behaviors in a sample of compulsive buyers.

	Males (*n* = 20)		Females (*n* = 151)		Test	P
SPQ Variables	Mean	SD	Mean	SD		
Alcohol	11.33	14.38	4.27	8.49	U = 513.5	0.044*
Shopping	32.80	6.80	36.43	6.94	U = 507	0.059
Food Bingeing	21.93	12.76	25.03	14.99	U = 646	0.451
Food Starving	10.87	8.7	9.49	7.72	U = 661.5	0.572
Submissive Helping	30.87	10.74	25.25	9.75	t = 2.48	0.043*
Dominant Helping	22.80	11.15	16.41	10.70	t = 2.14	0.035*
Tobacco	2.13	5.72	4.41	10.05	U = 706	0.814
Gambling	12.00	11.03	3.52	5.94	U = 274	<0.001**
Drugs	2.80	8.70	1.29	4.49	U = 678.5	0.560
Sex	15.27	14.36	4.35	7.37	U = 334	<0.001**
Work	22.27	10.40	15.92	8.83	t = 2.50	0.014*
Relationship Dominant	18.33	14.32	12.04	11.60	U = 530	0.090
Relationship Submissive	17.32	10.84	9.96	9.01	U = 420	0.009*
Caffeine	8.67	12.01	6.1	7.83	U = 607.5	0.297
Prescription Drugs	8.73	13.38	7.89	9.97	U = 699.5	0.806
Exercise	14.07	11.37	8.81	8.17	U = 519	0.075

U = Mann Whitney U

t = T-Tests

*p < .05

**p < .01.

### Binary Logistic Regression

The overall model fit was significant *χ*^2^(6) = 43.39, *p* < .001, with an overall classification accuracy of 94.9% (Table 4). The results of the binary logistic regression found that men were more likely to report being non-heterosexual, more likely to be diagnosed with intermittent explosive disorder and more likely to be diagnosed with a sexual addiction. Men were also more likely to report fewer years of formal education, and less likely to report problems with shopping as measured by the SPQ.

**Table 4:** Backward binary logistic regression with gender coded (0 = female, 1 = male), with variables associated *p* < .10 entered as predictors.

Variables	Wald χ2	P	Exp (B)	95% for Exp (B)	
				Lower	Upper
Non-Heterosexual	7.00	0.008	24.31	2.28	258.72
Years of Formal Education	3.86	0.049	0.73	0.53	2.038
Sex Addiction	8.83	0.003	59.26	4.01	874.77
Intermittent Explosive Disorder	4.20	0.041	20.32	1.14	362.63
SPQ shopping	5.73	0.017	0.86	0.76	0.97
Constant	3.20	0.074	244.05		

## Discussion

The results of our research found that while men and women did not differ in compulsive buying severity, there were important qualitative differences. Specifically, male compulsive buyers were more likely to be non-heterosexual, report fewer years of formal education, score higher on the shopping subscale of the SPQ and more likely to be diagnosed with sex addiction and intermittent explosive disorder. As such, the present research adds to the growing understanding of the potential gender differences in CBD. Indeed, despite the increasing empirical literature on compulsive buying, there has been a paucity of research examining the potential gender differences in this clinical disorder.

Consistent with the literature, our sample consisted mostly of women with men making up 11.7% (*n* = 20) of the sample despite no difference in severity of compulsive buying. This is in contrast to existing literature, in which women tend to report greater severity of compulsive buying [21]. A potential reason for this discrepancy may be due to the low number of males in the published literature, including the present study. Interestingly, however, male compulsive buyers were more likely to present with higher levels of psychopathology. This finding may be due in part that excessive shopping behavior is more acceptable in women than in men in certain cultures. Thus, it might be easier for women to accept that there is something wrong with their shopping behavior, and seek appropriate treatment while men tend to deny the problem. However, this is merely speculative and in need of empirical investigation.

Notably, while there were no differences in compulsive buying severity, the results of the binary logistic regression found that men reported less severe shopping behaviors as measured by the SPQ. A potential reason for this finding could be due that while the CBS measures the adverse consequences and dysfunctionality of compulsive buying, specifically as it relates to loss of control with finances (e.g., *“I have bought things thought I couldn’t afford them*”) the SPQ shopping subscales focuses more on the enjoyment associated with shopping (e.g., *“I tend to use shopping as both a comfort and a strength as both a comfort and a strength even when I do not need anything”*). Previous research suggests that emotions and identity-related dimensions of shopping, which are assessed by SPQ are more relevant for women than men [22,23,24]. Thus, given the present research used a treatment seeking sample, it is not surprising that while men and women did not report differences in harms related to compulsive shopping, men reported enjoying shopping less than women.

The results of the present study suggest that male compulsive buyers are likely to seek treatment when experiencing greater levels of distress and impairment in functioning caused by either compulsive buying or co-morbid psychopathology. Of potential importance is the finding that intermittent explosive disorder was more common in men. This may suggest that male compulsive buyers have more difficulties controlling their impulses, potentially explaining the higher rates of comorbidity with disorders related to dysfunctions in impulse control. Further, the reduced ability to control impulses, especially aggression may lead to further impairments in functioning for male compulsive buyers, thus increasing the likelihood for seeking treatment.

The findings of the present research has important implications for the treatment of compulsive buyers. It would behoove treatment providers of compulsive buying disorder to assess co-morbid psychiatric disorders, given that almost everyone in the sample met criteria for at least one disorder. This can be done using a brief semi-structured interview such as the M.I.N.I. [15] or using a variety of psychometrically sound screening instruments such as the Beck Depression Inventory—II [25] and the Beck Anxiety Inventory [26]. The rates of co-morbidity in this sample are in line with previous literature, which found co-morbid rates as high as 90% [27]. A potential reason for the high rate of co-morbidity found in compulsive buying maybe due to the high levels of impairments associated with this disorder. Compulsive buying results in significant financial and social problems [13,28]. In turn, these impairments may exacerbate or lead to co-morbid psychiatric disorders, specifically depression and anxiety. The high rates of co-morbidity in individuals may also be due to compulsive buying being a self-medication strategy. In other words, akin to gambling [29], and sex addiction [30], compulsive buying may act as a maladaptive coping mechanism and helps to alleviate emotional distress in compulsive buyers.

Assessment of psychiatric comorbidities may especially be pertinent in male compulsive buyers, as they are more likely to report greater psychopathology than their female counterparts. Treating co-morbid psychiatric disorders in addition to compulsive buying is likely to lead to better treatment outcomes compared to treating compulsive buying alone. For example, there is substantial evidence that integrated treatments that addresses both to substance use disorders and co-morbidities reduces the severity of both disorders, improve treatment outcomes, and reduce health care costs [31,32]. Further, the current findings suggest that the treatment of male compulsive buyers needs to take gendered experiences into account, specifically in regards to psychiatric co-morbidities. A model to do so may be found in the transdiagnostic treatment of anxiety disorders [33]. Applied to the treatment of male compulsive buyers, interventions may wish to target psychological processes underlying co-morbid disorders or transdiagnostic processes (e.g., impulsivity), rather than targeting the symptoms of specific disorders, such as reducing the number of items purchased. Indeed, targeting transdiagnostic processes will likely lead not only to improvements in compulsive buying, but also improvements in the co-morbid disorders.

Several limitations of the present research should be noted. First, the relatively small number of men in the present sample resulted in wide confidence intervals. However, the statistical analyses used in the present research (e.g., binary logistic regression) are robust to unequal sample sizes, restoring some confidence in our findings. Second, there are likely to be other important gender differences in compulsive buying on factors that were not assessed in the present research. For example, psychological constructs such as impulsivity and emotional dysregulation may be important gender differences in compulsive buyers. Lastly, given the exploratory nature of the manuscript, the results herein should be viewed as preliminary evidence for the potential gender differences in compulsive buying disorder.

## Conclusion

Compulsive buying has been described since the genesis of psychiatry. However, it is only recently that empirical investigations have shed light into the etiology, demographic and clinical correlates of compulsive buying. Herein, we add to the growing understanding of compulsive buying disorder by delineating gender differences in this common clinical disorder. The results suggest that treatment of compulsive buying needs to take into account the gendered differences between male and female compulsive buyers. Indeed, male compulsive buyers are significantly more likely to present with higher psychopathology, which may have important implications in the prognosis and treatment of compulsive buying. Taking into account gender differences may assist in the development of more individualized treatment of compulsive buying disorder, and ultimately increase the rate of treatment success.
